# Safety and efficacy of RT234 vardenafil inhalation powder on exercise parameters in pulmonary arterial hypertension: phase II, dose-escalation study design

**DOI:** 10.1186/s12931-022-02262-9

**Published:** 2022-12-17

**Authors:** Raymond L. Benza, Veronica Franco, Mandar A. Aras, Leslie Spikes, Daniel Grinnan, Carol Satler

**Affiliations:** 1 Division of Cardiovascular Medicine, The Ohio State University Wexner Medical Center, 410 W 10th Ave, Columbus, OH 43210 USA; 2 Division of Cardiology, University of California San Francisco, San Francisco, CA USA; 3 University of Kansas Medical Center, Kansas City, KS USA; 4 Virginia Commonwealth University School of Medicine, Richmond, VA USA; 5 Respira Therapeutics, Inc., Boston, MA USA

**Keywords:** Cardiac output, Clinical trial protocol, Exercise test, Pulmonary arterial hypertension, Safety, Treatment efficacy, Vardenafil, Vascular resistance

## Abstract

**Background:**

Pulmonary arterial hypertension (PAH) is a progressive disease characterized by high mean pulmonary arterial pressure (≥ 20 mmHg) and remodeling of the vascular arteries. Approved therapies improve symptoms and delay clinical worsening in the long term, but they do not relieve acute exertional symptoms. RT234, a drug/device combination (Respira Therapeutics, Palo Alto, CA, USA) that delivers the phosphodiesterase 5 inhibitor vardenafil to the lungs via inhalation, has been shown to reduce pulmonary vascular resistance in patients with PAH. This study aims to evaluate whether RT234 can increase oxygen capacity during cardiopulmonary exercise testing (CPET) in patients with PAH.

**Methods:**

This prospective, multi-center, open-label, two-cohort, dose-escalation, phase IIb trial in patients with PAH will evaluate the safety and efficacy of RT234 in improving exercise parameters. The trial began in September 2020 and is expected to be completed by early 2024. Patients eligible for enrollment will have a right heart catheterization–confirmed diagnosis of PAH, a 6-minute walking distance of ≥ 150 m, a minute ventilation/carbon dioxide production slope of ≥ 36, and will be on up to three stable oral and/or inhaled (not parenteral) PAH-specific background therapies. The estimated sample size is 86 patients, who will be divided into two dose cohorts. Cohort 1 will receive 0.5 mg RT234, and cohort 2 will receive 1.0 mg RT234. Each cohort will contain two subgroups based on the number of PAH background medications (up to two vs three). The trial will assess patients’ changes from baseline in peak oxygen consumption (VO_2_) during CPET 30 minutes after a single dose of 0.5 mg or 1.0 mg RT234, the change in the 6-minute walking distance, and the pharmacokinetics and safety profile of single doses of RT234.

**Conclusion:**

This is the first trial involving an as-needed medication for PAH. The trial will provide insights into the safety and efficacy of as-needed RT234 in treating the acute symptoms of PAH during exercise and will inform the design of further trials.

*Trial registration number:* ClinicalTrials.gov identifier NCT04266197.

## Background

Pulmonary hypertension (PH) is a rare disease defined by abnormally high pulmonary arterial pressure and pulmonary vascular resistance [1, 2]. Group 1 PH, pulmonary arterial hypertension (PAH) [3], is a progressive disease characterized by a mean pulmonary artery pressure ≥ 20 mmHg, pulmonary capillary wedge pressure ≤ 15 mmHg, and pulmonary vascular resistance of ≥ 3 Wood units [4], as well as physiological changes to the pulmonary arteries [5]. As the disease progresses, exercise capacity declines and daily activities become more difficult [6].

There is currently no cure for PAH. The disease is managed by approved treatments, such as endothelin receptor agonists, phosphodiesterase 5 inhibitors (PDE5i), and prostacyclin analogs that reduce symptoms and slow disease progression over time [7]. Managing the acute symptoms induced by daily activities is an ongoing challenge for patients living with PAH [8, 9] because none of the approved treatments can be used for acute symptom relief. There is therefore an unmet clinical need for an as-needed (PRN) treatment for PAH that will reduce acute symptoms and allow patients to perform daily activities and exercise.

**Fig. 1:**
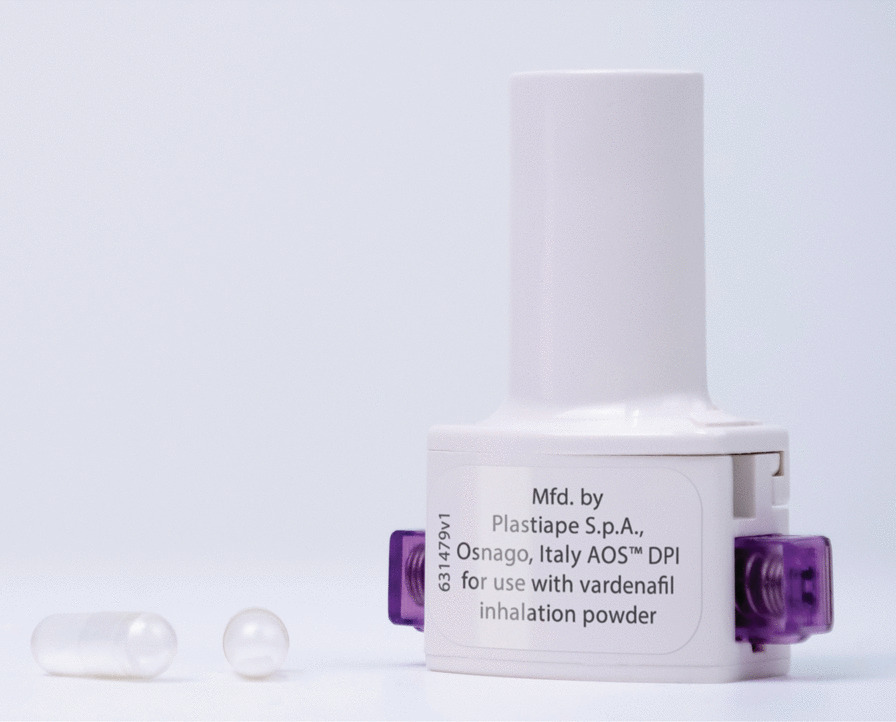
The Axial Oscillating Sphere (AOS) Dry Powder Inhaler (DPI) for inhalation of vardenafil. RT234 is a combination of vardenafil in powder form and the AOS DPI™ (Plastiape S.p.A., Osnago, Italy). Image copyright of Respira Therapeutics

Vardenafil is a PDE5i that has been shown to improve exercise capacity in patients with PAH [10]. Vardenafil has a slow dissociation rate (*K*_D_ 0.38 ± 0.07) [11] and rapid clearance (56 L h^–1^) [12], making it ideally suited to PRN use. An inhalable, powdered form of vardenafil has recently been developed, which is delivered via RT234 (Respira Therapeutics, Palo Alto, CA, USA), a drug/device combination product comprising powdered vardenafil hydrochloride and the Axial Oscillating Sphere Dry Powder Inhaler™ (DPI; Plastiape S.p.A., Osnago, Italy (Fig. 1)). A phase I study, RT234-CL101, has been completed for RT234 [13], which compared the pharmacokinetic (PK) profiles of RT234 and oral vardenafil in healthy volunteers. The maximum concentration (C_max_) of vardenafil was reached rapidly (time to C_max_ [T_max_] = 2 minutes), there were no serious treatment-emergent adverse events (TEAEs), and the drug was well tolerated.

RT234-CL202 is a phase IIb study that will investigate acute changes in exercise capacity and exertional symptoms, as assessed by cardiopulmonary exercise testing (CPET) and the 6-minute walking test (6MWT), following RT234 dosing in patients with PAH on stable, disease-specific background therapy. The study will evaluate the acute effect of single doses of 0.5 mg and 1.0 mg RT234 on CPET parameters and the change in 6-minute walking distance (6MWD) in two sequential cohorts. The adverse event profile and acute physical and cardiac effects of RT234 will be determined, as well as the PK parameters of vardenafil and the relationship between vardenafil exposure and changes in CPET parameters and the 6MWD. This study will provide important insights into the effectiveness of RT234 for improving exercise capacity in patients with PAH on stable background therapy.

## Methods

### Study design

This prospective, multi-center, open-label, two-cohort, dose-escalation, phase IIb trial in patients with PAH on ≤ 3 stable, disease-specific PAH background oral and/or inhaled therapies will evaluate the safety and efficacy of RT234 on exercise parameters. It is estimated that this trial will be conducted at up to 25 sites in the United States and Serbia. The trial began in September 2020 and is expected to be completed by early 2024. All CPET data collected during the trial will be transmitted electronically to a central facility, the CPET Core Laboratory, where they will be regularly reviewed. The trial will be conducted in accordance with the Declaration of Helsinki and the Good Clinical Practice Guidelines of the International Conference on Harmonisation, along with all local regulatory requirements. The informed consent document and the protocol have been or will be reviewed and approved by each investigator’s designated Institutional Review Board/Independent Ethics Committee, and by the sponsor. The trial is registered at ClinicalTrials.gov (identifier NCT04266197).

At each site, the investigator will obtain and document written informed consent for each patient and will determine the eligibility of patients before enrolling them in the trial. Patients will be divided into two successive cohorts, cohort 1 (receiving 0.5 mg RT234) and cohort 2 (receiving 1.0 mg RT234), with up to 86 patients in total. Each dose cohort will be further divided into two subgroups as defined by the number of background medications (i.e., up to two vs three). There will be up to 17 patients in each dose cohort using up to two background medications and up to 26 patients using three background medications. A Safety Monitoring Committee will independently review all safety and efficacy data from the trial once they have been collected. Data for patients who discontinue the study and provide an explanation will be summarized and listed.

### Study population

Each patient’s eligibility will be reviewed by the sponsor’s medical monitor before and after the baseline CPET. To be eligible for inclusion in the trial, patients must be between 18 and 80 years of age, inclusive; have a diagnosis of right heart catheterization–confirmed PAH with WHO/New York Heart Association functional class II–IV symptomatology; have a body mass index ≤ 35.9 kg m^–2^; be on stable PAH disease-specific background therapy of up to three oral and/or inhaled therapies; have a 6MWD of ≥ 150 m; and have minute ventilation (VE)/carbon dioxide production (VCO_2_) slope of ≥ 36 (Table 1). The sponsor’s chief medical officer or designated medical monitor must approve RT234 dosing. Patients will be excluded from the trial if they have baseline systemic hypotension, defined as mean arterial pressure < 50 mmHg or systolic blood pressure (SBP) < 90 mmHg, or a history of uncontrolled hypertension, defined as SBP > 175 mmHg or sitting diastolic blood pressure > 110 mmHg; use parenteral PAH medications; or have evidence or history of left-sided heart disease and/or clinically significant cardiac disease (Table 1).

**Table 1:** Patient inclusion and exclusion criteria

**Inclusion criteria**
• ≥ 18 to ≤ 80 years of age
• BMI ≤ 35.9 kg m^–2^*
• Diagnosis of documented and RHC–confirmed PAH in any of the following: • Idiopathic, primary, or familial PAH ﻿ OR • PAH associated with connective tissue diseases OR • PAH associated with: HIV; simple, congenital systemic–to–pulmonary shunts ≥ 1-year post–surgical repair; exposure to drugs, chemicals, and toxins
• The patient must have had ventilation/perfusion scan, computerized tomography angiogram, or pulmonary arteriogram that rules out chronic thromboembolic pulmonary hypertension
• Previous PAH diagnosis with the following conditions: • Stable PAH without significant adjustments of disease–specific background PAH therapy ≥ 3 months before the CPET procedure AND • If on corticosteroids, has been receiving a stable dose of ≤ 20 mg per day of prednisone (or equivalent dose of other corticosteroid) for ≥ 30 days before the baseline CPET
• PFT within 6 months before commencement of screening, or during the screening period for this study, that fulfills the following: • FEV_1_ ≥ 60% predicted • FVC ≥ 60% predicted • FEV_1_/FVC ≥ 60%
• RHC performed and documented before screening that is consistent with the diagnosis of PAH, meeting all the following criteria: • mPAP ≥ 20 mmHg (at rest) AND • PCWP or LVEDP of ≤ 12 mmHg if PVR is ≥ 300 to < 500 dyn·s cm^– 5^, or PCWP or LVEDP ≤ 15 mmHg if PVR is ≥ 500 dyn·s cm^–5^ and, if PCWP is not available, then mLAP or LVEDP ≤ 15 mmHg or ≤ 12 mmHg in the absence of left atrial obstruction AND • PVR > 3 Wood units or > 240 dyn·s cm^–5^
• WHO/NYHA functional class II–IV symptomatology
• On stable^†^ PAH disease–specific background therapy of ≤ three oral therapies and/or inhaled therapy
• Able to walk ≥ 150 m on the 6MWT^‡^
• Has a VE/VCO_2_ slope ≥ 36 during the baseline CPET^§^
• Maximal effort on the baseline CPET must reach a peak RER of ≥ 1.0
• If the patient is taking the following concomitant medications, which may affect PAH, the patient must be on a stable therapeutic dose for ≥ 1 month before the screening, and the dosage must be maintained throughout the study: • Vasodilators • Digoxin • L–arginine supplement • Anticoagulants (anticoagulation status should be maintained/stable in the therapeutic range for ≥ 1 month before the screening)
**Exclusion criteria**
• Baseline systemic hypotension, defined as MAP < 50 mmHg or SBP < 90 mmHg at screening
• History of chronic uncontrolled asthma
• Patients who are unable or may find it difficult to use an inhaler device
• Requirement for intravenous inotropes within 30 days before the baseline CPET procedure
• Use of parenteral PAH medications
• Use of ricioguat as background PAH therapy ≤ 1 month before initiating screening or during the study through the end of visit 4
• Use of oral, topical, or inhaled nitrates within 2 weeks before the baseline CPET procedure
• Has history of or current uncontrolled systemic hypertension as evidenced by sitting SBP > 175 mmHg or sitting DBP > 110 mmHg at screening
• Portopulmonary hypertension, portal hypertension, or chronic liver disease determined to be Child–Pugh B or C
• Evidence or history of left–sided heart disease and/or clinically significant cardiac disease
• History of atrial septostomy
• History of known uncorrected right–to–left shunt; clinically relevant, persistent patent foramen ovale; or known Eisenmenger’s physiology
• Paroxysmal or uncontrolled atrial fibrillation
• Chronic renal insufficiency
• Serum alanine aminotransferase or aspartate aminotransferase that is ≥ 3x the upper limit of the normal range
• Platelets < 50,000 µL^–1^ at screening
• Hemoglobin concentration < 9 g dL^–1^ at screening

*6MWT* 6-minute walking test, *BMI* body mass index, *CPET* cardiopulmonary exercise testing, *DBP* diastolic blood pressure, *FEV*_*1*_ forced expiratory volume in 1 second, *FVC* forced expiratory vital capacity, *LVEDP* left ventricular end-diastolic pressure, *MAP* mean arterial pressure, *mLAP* mean left atrial pressure, *mPAP* mean pulmonary arterial pressure, *NYHA* New York Heart Association, *PAH* pulmonary arterial hypertension, *PCWP* pulmonary capillary wedge pressure, *PFT* pulmonary function testing, *PVR* pulmonary vascular resistance, *RER* respiratory exchange ratio, *RHC* right heart catheterization, *SBP* systolic blood pressure, *VE/VCO*_*2*_ ventilation/carbon dioxide production, *WHO* World Health Organization

*To consider individuals with BMI > 36 kg m^–2^, contact the sponsor Medical Director

^†^Stable is defined as no change in PAH-specific drug therapy within 3 months of screening visit 1 and for the duration of the study, and no change in dose of PAH-specific drug(s) within 1 month of screening

^‡^6MWT distance will be determined using the mean of the two 6MWT results done between visits 1 and 2, ≥ 2 hours apart if conducted on the same day, or ≥ 2 days apart

^§^As assessed by the CPET Core Laboratory

### Treatment protocol

**Fig. 2:**
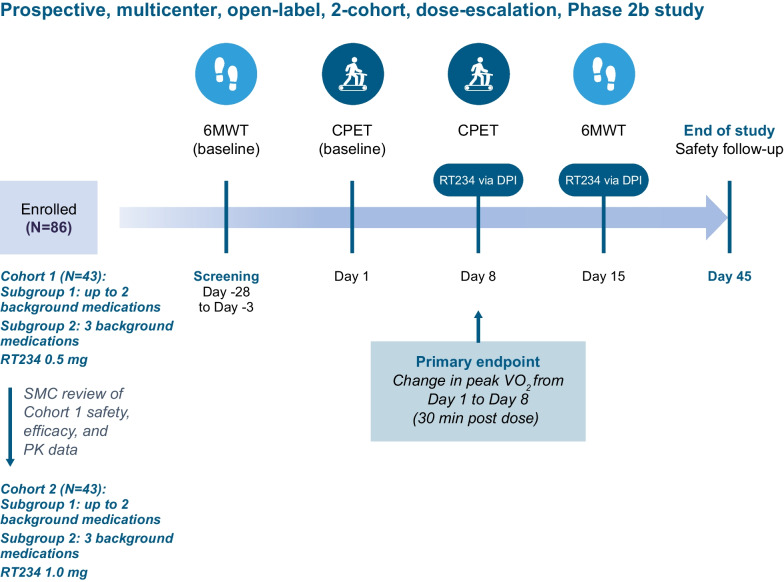
Study design. Patients with pulmonary arterial hypertension will be assigned to two successive cohorts to receive one dose of RT234 (0.5 mg for cohort 1 and 1.0 mg for cohort 2) via the Dry Powder Inhaler (DPI; Plastiape S.p.A., Osnago, Italy). Screening 6-minute walking tests (6MWT) to determine baseline 6-minute walking distance will be conducted between days − 28 and − 3 of the study (visit 1). On day 1 (visit 2), patients will undergo baseline cardiopulmonary exercise testing (CPET) to measure peak oxygen consumption (VO_2_). On day 8 (visit 3), patients will receive 0.5 mg (cohort 1) or 1.0 mg (cohort 2) RT234 and will perform CPET 30 minutes post dose. On day 15 (visit 4), patients will receive 0.5 mg (cohort 1) or 1.0 mg (cohort 2) RT234 and will perform the 6MWT 30 minutes post dose. Blood samples will be collected at visits 3 and 4 before RT234 dosing; at 3, 15, and 30 minutes post dose; immediately at the end of the exercise period; and at 45, 75, 120, 180, and 240 minutes post dose for pharmacokinetic (PK) measurements. Patients will be monitored for 240 minutes post dose at visits 3 and 4. Follow-up will be conducted by telephone on day 45 (visit 5). Safety will be monitored throughout the study by the Safety Monitoring Committee (SMC)

**Fig. 3:**
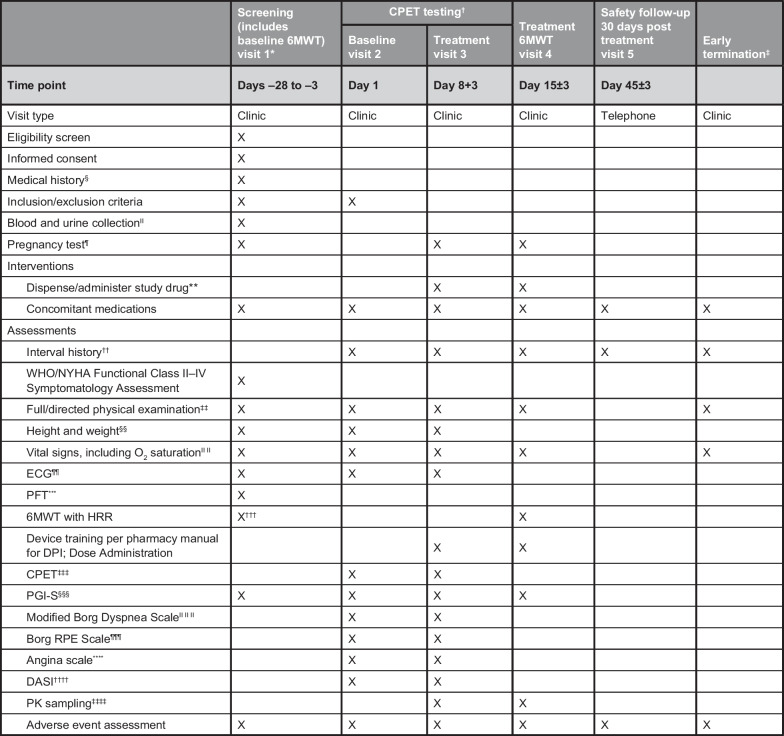
Schedule of events. Study assessments will be performed over 45 days for each cohort. The schedule lists all the interventions, tests, and pre- and post-test assessments that will be performed at screening (days − 28 to − 3), cardiopulmonary exercise testing (CPET) at baseline (day 1), treatment (days 8 and 15), and safety follow-up (day 45) visits. Adverse event assessments and interval history reviews will be conducted at each visit. *6MWT* 6-minute walking test, *6MWD* 6-minute walking distance, *BP* blood pressure, *DASI* Duke Activity Status Index, *ECG* electrocardiogram, *HR* heart rate, *HRR* heart rate recovery, *NYHA* New York Heart Association, *O*_*2*_ oxygen, *PAH* pulmonary arterial hypertension, *PFT* pulmonary function testing, *PGI-S* Patient Global Impression of Severity, *PK* pharmacokinetic, *RPE* rating of perceived exertion, *SPO*_*2*_ peripheral oxygen saturation, *WHO* World Health Organization. *Potential patients will be screened approximately 3 days (≤ 28 days) before baseline CPET visit 2. ^†^Patients will be scheduled for treatment CPET visit 3, approximately 7 days after the baseline CPET visit 2. ^‡^If a patient prematurely discontinues the study any time before dosing or treatment CPET visit 3 because of an adverse event or patient safety concern, the patient should return to the clinic as soon as possible for an early termination study visit. ^§^Medical history will include a detailed PAH disease-specific medical history. ^II^Clinical laboratory tests may be repeated at the baseline CPET visit 2 if screening laboratory results are deemed clinically significant and warrant repeat testing. Clinical laboratory includes hematology, chemistry, and urinalysis. Prothrombin time international normalized ratio will also be tested in patients taking oral vitamin K antagonists. ^¶^Urine or serum pregnancy test at screening visit 1 required and a urine or serum pregnancy test at visit 3 and visit 4. **Either a single 0.5-mg dose (cohort 1) or a single 1.0-mg dose (cohort 2) of RT234 will be administered 30 minutes before CPET at visit 3 and 30 minutes before the 6MWT at visit 4. ^††^Interval history will include any signs, symptoms, or events experienced by the patient since the previous study visit. ^‡‡^Physical examinations at screening will be complete; all other physical examinations will be symptom directed. ^§§^Height will be obtained only at screening visit 1. ^II II^Resting vital signs (BP, HR, respiration rate, body temperature, and pulse oximetry [SPO_2_]) after resting for 5 minutes (sitting). Vital signs should be taken before any blood draw. BP, HR, and SPO_2_ will also be measured during CPET and into recovery (HR via continuous ECG monitoring during CPET and for 6 minutes post CPET, BP assessment every 2 minutes during CPET and for 6 minutes post CPET, and SPO_2_ assessment every minute during CPET and for 6 minutes post CPET) and at treatment 6MWT visit 4 (before RT234 dosing; 5 and 15 minutes post dose before 6MWT at 30 minutes post dose; and at 60 and 120 minutes post dose). ^¶¶^Continuous ECG monitoring will be collected during CPET and for 6 minutes post CPET. ***PFT may be completed at screening and/or baseline to confirm eligibility if historical PFT results within 6 months before screening are not available. ^†††^During the screening period, two 6MWTs will be performed at least 2 days apart, and the mean of the distance walked will be used for eligibility. If tolerable for the patient, the two 6MWTs at screening can be performed on the same day after a minimum of 2 hours between each test. The two screening 6MWDs must have a relative difference (i.e., [absolute difference between the two 6MWDs] / [mean of the two 6MWDs]) of ≤ 15%. If the difference is > 15%, a third 6MWT will be done during screening. If after the third 6MWT the relative distance is still > 15%, the Sponsor Medical Monitor will be contacted. The two longest 6MWDs will be averaged to determine the baseline 6MWD. ^‡‡‡^ The CPET assessments conducted at Visits 2 (pre dose) and 3 (post dose) will be conducted at approximately the same time of day (within 2 hours). ^§§§^PGI-S before CPET and at 2 minutes cool down off treadmill but still wearing the mask, then at 5-minute intervals: 7, 12, and 17 minutes with the mask off. For the 6MWT, PGI-S will be taken before and at 2 minutes after completion of the test. ^II II II^Modified Borg Dyspnea Scale assessment every minute during CPET and for 6 minutes post CPET. ^¶¶¶^Borg RPE Scale assessment every 2 minutes during CPET and for 6 minutes post CPET. ****Angina Scale assessment every 2 minutes during CPET and for either 6 minutes post CPET or, if indicated, until symptom recovery. ^††††^DASI assessment 10 minutes post CPET. ^‡‡‡‡^PK samples will be collected at visits 3 and 4 before RT234 dosing; at 3, 15, and 30 minutes post dose; immediately at the end of the exercise period; and at 45, 75, 120, 180, and 240 minutes post dose

Once patient eligibility has been confirmed, the study will begin with the screening visit(s) (visit 1), which will occur between 28 and 3 days before the CPET baseline visit (Fig. 2). The screening visit(s) will include two 6MWTs performed at least 2 days apart or, if tolerable to the patient, at least 2 hours apart on the same day (this will account for the learning effect associated with the 6MWT [14]; the relative difference between the two measurements must be ≤ 15%). The two screening 6MWDs will be averaged to determine the baseline 6MWD. At the CPET baseline visit (visit 2; day 1), enrolled patients will return to the testing center for pre-test assessments, CPET, and post-test assessments (Fig. 3). Eligible patients whose baseline CPETs are deemed evaluable by the CPET Core Laboratory will enter the treatment period (visits 3 and 4) for dosing with RT234 and post-dose CPET and 6MWT. At the CPET treatment visit (visit 3; day 8), patients will return to the testing facility for pre-CPET assessments and a single dose of RT234 (0.5 mg for cohort 1 and 1.0 mg for cohort 2), which will be administered with the aid of the study team. CPET will be performed 30 minutes post dose, at approximately the same time of day at each visit (within 2 hours), followed by post-test assessments. At the 6MWT treatment visit (visit 4; day 15), patients will undergo pre-6MWT assessments and receive a single dose of RT234. Patients will perform a 6MWT 30 minutes post dose and will then undergo post-test assessments. Plasma samples for PK analyses will be collected before and after RT234 dosing at visits 3 and 4. RT234 will only be administered to patients during visits 3 and 4 (Fig. 1); patients will not use RT234 outside these two visits. Patients will remain at the clinic for PK sampling and safety monitoring for 4 hours once all tests have been completed during visits 3 and 4, and safety monitoring will continue for 30 days after visit 4. Visit 5 (day 45 ± 3-day window) will involve a follow-up telephone call assessment of safety.

### Outcome measures and endpoints

The primary efficacy endpoint is the change from baseline in peak oxygen consumption (VO_2_) measured during CPET performed 30 minutes after a single dose of 0.5 mg or 1.0 mg RT234. The secondary efficacy endpoints are change from baseline in 6MWD (mean of two 6MWDs at screening) to the 6MWD measured 30 minutes after RT234 dosing; change from baseline to post dose in the minute VE/VCO_2_ slope during CPET; change from baseline to post dose in the response of the partial pressure of end-tidal carbon dioxide apex to exercise (i.e., highest level during CPET); change from baseline to post dose in the duration of exercise during CPET; change from baseline to post dose in peak perceived dyspnea during CPET, as assessed by the Modified Borg Dyspnea Scale Score [15]; change from baseline to post dose in Patient Global Impression of Severity (PGI-S) for CPET (assessed before CPET and after a 2-minute cooldown after treadmill exercise while wearing mask, and then at 5-minute intervals [7, 12, and 17 minutes] without a mask); and changes from screening to post dose in PGI-S for 6MWT (assessed before the 6MWT and at 2 minutes after completion of the 6MWT).

The assessment of safety will involve evaluating the adverse event profile and acute physical and cardiac symptoms of single doses (0.5 or 1.0 mg) of RT234. The incidence and severity of TEAEs will be summarized by Medical Dictionary for Regulatory Activities (current version) System Organ Class and Preferred Term, National Cancer Institute Common Terminology Criteria for Adverse Events (current version) grade, and causality (attribution to study treatment and related/not related to investigational medicinal product). Changes in vital signs (blood pressure [BP], heart rate, respiratory rate, body temperature, and pulse oximetry) will be monitored, and physical examinations and 12-lead electrocardiograms (ECGs) will be conducted to measure the change from baseline to post dose.

Exploratory endpoints include the proportion of patients with improvement in risk category (low, intermediate, or high) for the VE/VCO_2_ slope criteria (defined in the 2015 European Society of Cardiology/European Respiratory Society Guidelines for the Diagnosis and Treatment of Pulmonary Hypertension [1]) during CPET from baseline to post dose, and changes in the following additional CPET parameters from baseline to post dose, according to the recommendations of the American Heart Association [16]: ECG response to exercise, VO_2_ at ventilatory threshold, change in respiratory exchange ratio at peak VO_2_, systolic BP response to exercise, pulse oximetry response to exercise, the non-peak Modified Borg Dyspnea Scale Score [15] throughout exercise (at every non-peak minute during CPET and for 6 minutes post CPET), the Borg Rating of Perceived Exertion Scale [17] throughout exercise (every 2 minutes during CPET and recovery), the Duke Activity Status Index [18] and the 4-Point Angina Scale [19].

The PK parameters of vardenafil and the relationship between vardenafil exposure and changes in CPET parameters and 6MWD will also be determined. Plasma samples will be collected at visits 3 and 4 before RT234 dosing; at 3, 15, and 30 minutes post dose; immediately at the end of the exercise period; and at 45, 75, 120, 180, and 240 minutes post dose. Vardenafil concentrations will be measured in the PK samples as described previously [13]. Estimators of PK parameters will include T_max_, C_max_, area under the curve from time 0 to the last measurable concentration (AUC_0–Last_), AUC from time 0 to infinity (AUC_0–Inf_), and half-life (t_1/2_). For CPET, exposure–response analysis will be based on AUC_0–Last_ and change from baseline in peak VO_2_ measured during CPET 30 minutes after RT234 dosing; for the 6MWT, exposure–response analysis will be based on AUC_0–Last_ and change from baseline 6MWD (mean of the screening 6MWDs) to the 6MWD 30 minutes after RT234 dosing.

### Statistical analysis

The estimated total sample size is 86 enrolled patients, with a 5% dropout rate and a computed sample size of 17 and 26 patients with up to two and three background medications, respectively, per dose cohort (0.5 and 1.0 mg). This was derived using a one-sample two-sided *t* test (0.05 significance level and 80% power) to test the null hypothesis of no change in peak VO_2_ from baseline to post dose assessed during CPET performed 30 minutes after RT234 dosing. The assumed mean change from baseline is 1.5 and 1.2 mL O_2_·kg^–1^·min^–1^ [20] for patients with up to two and three background medications, respectively, in each dose cohort. A standard deviation of 2 mL O_2_·kg^–1^·min^–‍1^ is assumed for each dose cohort and background medication group.

The primary efficacy analysis will be conducted on the modified intention-to-treat (mITT) analysis set, which comprises all treated patients with baseline and post-baseline assessment of peak VO_2_. This mITT analysis set will serve as the basis for all efficacy analyses. The analysis procedure will be repeated for the per-protocol analysis set (all mITT patients who do not experience any major protocol violations) using a one-sample *t* test (if peak VO_2_ follows a normal distribution) to test the two-sided null hypothesis (0.05 significance level) of no change in peak VO_2_. The 95% CI for peak VO_2_ mean change will be derived from the *t* test if the normality assumption is not rejected. If it is rejected, a one-sample Wilcoxon signed-rank test will be used to determine the median change in peak VO_2_ with a 95% CI estimate.

The secondary efficacy analyses will be conducted on the mITT analysis set and will be repeated with the per-protocol analysis set, as described for the primary efficacy analyses. Because this study is exploratory (open-label, uncontrolled, and non-randomized) and its focus is on dose-escalation safety and activity, formal inferential statistics are not planned, and multiplicity adjustment will not be performed.

Safety data will be summarized descriptively for the safety analysis set (all patients who receive at least one dose [partial or complete] of RT234; safety parameters will be summarized for the pooled background medication groups in each dose cohort) on the basis of TEAEs, clinical laboratory assessments, vital signs, weight, and physical examinations. Changes from baseline in these assessments will be summarized by cohort using standard descriptive statistics. TEAE incidence rates will be summarized by frequency and percentage for each cohort and overall.

Planned PK parameters (T_max_, C_max_, AUC_0–Last_, AUC_0–Inf_, and t_1/2_) will be calculated for treated patients with available PK measurements using the standard linear trapezoidal convention for all available drug concentration measurements. Standard descriptive statistics (mean, median, n, standard deviation, minimum, maximum, and coefficient of variation) will be used to summarize the PK parameters, along with the 90% CI, when appropriate.

## Discussion

RT234 is a first-in-class treatment for PAH that is designed to provide immediate relief for exertion-related symptoms. Current approved therapies for PAH are chronic treatments that require multiple doses to alleviate symptoms and delay disease progression. Moreover, despite ongoing advances in these therapies, patients continue to experience a significant reduction in cardiorespiratory fitness and exercise capacity [21]. These therapies are also associated with adverse effects that reduce patients’ quality of life, including nausea, headache, and flushing, as well as injection site pain and infection with infusion medications [22]. These adverse effects are due, in part, to the doses required for an effective local drug concentration in the pulmonary arteries. Vardenafil is a high-affinity inhibitor of PDE5 (half-maximal inhibitory concentration [IC_50_] 0.091 ± 0.031) [11], which, in combination with its slow dissociation rate, allows for lower concentrations of the drug to be used, reducing the chance of adverse effects. Furthermore, direct delivery to the lungs via RT234 reduces systemic exposure to the drug, as observed in RT234-CL101 [13]. This is supported by the results of the RT234-CL201 phase IIa study, in which the pulmonary vascular resistance/systemic vascular resistance ratio was reduced and stable mean systemic arterial pressure and systemic vascular resistance were maintained [23].

Two inhaled therapies have been approved to treat PAH, iloprost (Ventavis®, Actelion Pharmaceuticals, South San Francisco, CA, USA) and treprostinil (Tyvaso®, United Therapeutics, Silver Spring, MD, USA), which were developed as alternatives to intravenous therapies. However, these drugs have been formulated for use with a nebulizer, which is difficult and time consuming to operate [24, 25]. Two investigational dry powder formulations of treprostinil, Tyvaso DPI™ and Yutrepia™ (Liquidia, Morrisville, NC, USA), have also been approved or tentatively approved to treat PAH. These drugs have been developed as chronic treatments that will offer an alternative to nebulized treprostinil.

Inhaled PRN therapies have been shown to improve symptoms and exercise capacity in other diseases that impede exercise, for example, bronchodilators in patients with asthma [26] and oral nitroglycerin spray in patients with chronic angina [27]. Similarly, PRN therapy for PAH is expected to enable patients to exercise more easily, resulting in improved fitness and functional capacity [28]. RT234-CL202 is the first trial to test the acute effect of an inhaled PDE5i on exercise parameters in patients with PAH and is therefore the first to address the unmet need for a PRN therapy for PAH. This trial will extend the results of the phase I and IIa studies, which established that RT234 is ideally suited for use as a PRN treatment for PAH because it exhibits pulmonary selectivity, is rapidly absorbed and cleared [13], and is well tolerated by patients with PAH on background therapies [23]. Although these trials demonstrated that RT234 has an acceptable safety profile and improves hemodynamic parameters in patients with PAH, this phase IIb trial will specifically investigate the safety and efficacy of RT234 when used to acutely improve VO_2_ during exercise.

A strength of this study is that it is the first involving a PRN therapy for PAH. Additionally, to ensure that patients enrolled in the trial do not have reduced cardiopulmonary fitness due to factors other than PAH [29], individuals with confounding conditions such as hypotension, uncontrolled asthma, systemic hypertension, and cardiac disease are not eligible for this trial. The validity of the results will be ensured through the use of CPET to assess cardiopulmonary output because it allows for numerous clinically relevant measurements to be made simultaneously, including the change in VO_2_ [30]. The use of the 6MWT to assess exercise capacity will also contribute to validity because it has been used routinely in clinical trials for approved PAH therapies [31]. Although there are limitations to using the 6MWD as a clinical endpoint for long-term studies, it is a valid measure in short-term studies with small cohort sizes [29].

The main limitation of the study is the small number of patients, although the planned sample size was calculated to ensure sufficient power and to account for the effect of patient dropout, and the short duration of the trial will help to minimize the loss of patients. Along with this, the lack of a placebo arm and the open-label nature of the trial increase the risk of introducing bias. Finally, the trial excludes patients using intravenous PAH therapies because these patients generally have severe disease that may substantially limit their exercise capacity [32].

The results of the RT234-CL202 trial, expected by early 2024, will inform the design of phase III trials to determine the efficacy and safety of RT234 in patients with PAH. If these trials are successful, PRN RT234 is anticipated to provide acute improvements in patients’ capacities for exercise and physical activity and to reduce the exertional symptoms associated with physical activity, thereby improving the quality of life of those living with PAH.

## Data Availability

Data sharing is not applicable to this article as no datasets were generated or analyzed during the current study.

## Abbreviations

6MWD: 6-minute walking distance
6MWT: 6-minute walking test
AUC_0–Inf_: Area under the curve from time 0 to infinity
AUC_0–Last_: Area under the curve from time 0 to the last measurable concentration
BP: Blood pressure
C_max_: Maximum concentration
CPET: Cardiopulmonary exercise testing
DPI: Dry Powder Inhaler
ECG: Electrocardiogram
mITT: Modified intention-to-treat
PAH: Pulmonary arterial hypertension
PDE5i: Phosphodiesterase 5 inhibitors
PGI-S: Patient Global Impression of Severity
PH: Pulmonary hypertension
PK: Pharmacokinetic
PRN: As needed
SBP: Systolic blood pressure
t_1/2_: Half-life
TEAE: Treatment-emergent adverse event
T_max_: Time to maximum concentration
VCO_2_: Carbon dioxide production
VE: Ventilation
VO_2_: Peak oxygen consumption
WHO: World Health Organization

## References

[1] Galiè N, Humbert M, Vachiery JL, Gibbs S, Lang I, Torbicki A, et al; ESC Scientific Document Group. 2015 ESC/ERS Guidelines for the diagnosis and treatment of pulmonary hypertension: The Joint Task Force for the Diagnosis and Treatment of Pulmonary Hypertension of the European Society of Cardiology (ESC) and the European Respiratory Society (ERS): Endorsed by: Association for European Paediatric and Congenital Cardiology (AEPC), International Society for Heart and Lung Transplantation (ISHLT). Eur Heart J. 2016;37:67–119. 10.1093/eurheartj/ehv317 26320113

[2] Chemla D, Castelain V, Herve P, Lecarpentier Y, Brimioulle S (2002). Haemodynamic evaluation of pulmonary hypertension. Eur Respir J.

[3] Simonneau G, Robbins IM, Beghetti M, Channick RN, Delcroix M, Denton CP (2009). Updated clinical classification of pulmonary hypertension. J Am Coll Cardiol.

[4] Simonneau G, Montani D, Celermajer DS, Denton CP, Gatzoulis MA, Krowka M (2019). Haemodynamic definitions and updated clinical classification of pulmonary hypertension. Eur Respir J.

[5] Lai YC, Potoka KC, Champion HC, Mora AL, Gladwin MT (2014). Pulmonary arterial hypertension: the clinical syndrome. Circ Res.

[6] Matura LA, McDonough A, Carroll DL (2014). Health-related quality of life and psychological states in patients with pulmonary arterial hypertension. J Cardiovasc Nurs.

[7] Barst RJ, Gibbs JSR, Ghofrani HA, Hoeper MM, McLaughlin VV, Rubin LJ (2009). Updated evidence-based treatment algorithm in pulmonary arterial hypertension. J Am Coll Cardiol.

[8] Frumkin LR (2012). The pharmacological treatment of pulmonary arterial hypertension. Pharmacol Rev.

[9] The voice of the patient. A series of reports from the U.S. Food and Drug Administration’s (FDA’s) Patient Focused Development initiative: pulmonary arterial hypertension. https://www.fda.gov/media/90479/download. Accessed 9 Feb 2021.

[10] Jing ZC, Yu ZX, Shen JY, Wu BX, Xu KF, Zhu XY (2011). Efficacy and Safety of Vardenafil in the Treatment of Pulmonary Arterial Hypertension (EVALUATION) Study Group. Vardenafil in pulmonary arterial hypertension: a randomized, double-blind, placebo-controlled study. Am J Respir Crit Care Med.

[11] Blount MA, Beasley A, Zoraghi R, Sekhar KR, Bessay EP, Francis SH (2004). Binding of tritiated sildenafil, tadalafil, or vardenafil to the phosphodiesterase-5 catalytic site displays potency, specificity, heterogeneity, and cGMP stimulation. Mol Pharmacol.

[12] Levitra®. https://www.accessdata.fda.gov/drugsatfda_docs/label/2008/021400s011lbl.pdf. Accessed 25 Mar 2022.

[13] Eldon MA, Parsley EL, Maurer M, Tarara TE, Okikawa J, Weers JG (2021). Safety, tolerability, and pharmacokinetics of RT234 (vardenafil inhalation powder): a first-in-human, ascending single- and multiple-dose study in healthy subjects. J Aerosol Med Pulm Drug Deliv.

[14] Wu G, Sanderson B, Bittner V (2003). The 6-minute walk test: how important is the learning effect?. Am Heart J.

[15] Mahler DA, Horowitz MB (1994). Perception of breathlessness during exercise in patients with respiratory disease. Med Sci Sports Exerc.

[16] Myers J, Arena R, Franklin B, Pina I, Kraus WE, McInnis K (2009). Recommendations for clinical exercise laboratories: a scientific statement from the American Heart Association. Circulation.

[17] Borg GA (1982). Psychophysical bases of perceived exertion. Med Sci Sports Exerc.

[18] Phillips L, Wang JW, Pfeffer B, Gianos E, Fisher D, Shaw LJ (2011). Clinical role of the Duke Activity Status Index in the selection of the optimal type of stress myocardial perfusion imaging study in patients with known or suspected ischemic heart disease. J Nucl Cardiol.

[19] Myers JN (1994). Perception of chest pain during exercise testing in patients with coronary artery disease. Med Sci Sports Exerc.

[20] Puente-Maestu L, Palange P, Casaburi R, Laveneziana P, Maltais F, Neder JA (2016). Use of exercise testing in the evaluation of interventional efficacy: an official ERS statement. Eur Respir J.

[21] Yorke J, Deaton C, Campbell M, McGowen L, Sephton P, Kiely DG (2018). Symptom severity and its effect on health-related quality of life over time in patients with pulmonary hypertension: a multisite longitudinal cohort study. BMJ Open Respir Res.

[22] Galiè N, Corris PA, Frost A, Girgis RE, Granton J, Jing ZC (2013). Updated treatment algorithm of pulmonary arterial hypertension. J Am Coll Cardiol.

[23] Keogh A, Dwyer N, Kotlyar E, Kaye D. Acute hemodynamic improvement in chronic pulmonary arterial hypertension on dual therapy following RT234 inhalation. In: Pulmonary Hypertension Association: June 10–12, 2022; Atlanta, GA.

[24] Olschewski H, Simonneau G, Galie N, Higenbottam T, Naeije R, Rubin LJ (2002). Aerosolized Iloprost Randomized Study Group. Inhaled iloprost for severe pulmonary hypertension. N Engl J Med.

[25] Parker DK, Shen S, Zheng J, Ivy DD, Crotwell DN, Hotz JC (2017). Inhaled treprostinil drug delivery during mechanical ventilation and spontaneous breathing using two different nebulizers. Pediatr Crit Care Med.

[26] Ostrom NK, Taveras H, Iverson H, Pearlman DS (2015). Novel albuterol multidose dry powder inhaler in patients with exercise-induced bronchoconstriction: a single-dose, double-blind, randomized, 2-way crossover study. Respir Med.

[27] Kimchi A, Lee G, Amsterdam E, Fujii K, Krieg P, Mason DT (1983). Increased exercise tolerance after nitroglycerin oral spray: a new and effective therapeutic modality in angina pectoris. Circulation.

[28] Buys R, Avila A, Cornelissen VA (2015). Exercise training improves physical fitness in patients with pulmonary arterial hypertension: a systematic review and meta-analysis of controlled trials. BMC Pulm Med.

[29] Gaine S, Simonneau G (2013). The need to move from 6-minute walk distance to outcome trials in pulmonary arterial hypertension. Eur Respir Rev.

[30] Guazzi M, Arena R, Halle M, Piepoli MF, Myers J, Lavie CJ (2016). 2016 focused update: clinical recommendations for cardiopulmonary exercise testing data assessment in specific patient populations. Circulation.

[31] Sung SH, Yeh WY, Chiang CE, Huang CJ, Huang WM, Chen CH (2021). The prognostic significance of the alterations of pulmonary hemodynamics in patients with pulmonary arterial hypertension: a meta-regression analysis of randomized controlled trials. Syst Rev.

[32] Vachiéry JL, Simonneau G (2010). Management of severe pulmonary arterial hypertension. Eur Respir Rev.

